# Eﬄux-mediated resistance to a benzothiadiazol derivative effective against *Burkholderia cenocepacia*

**DOI:** 10.3389/fmicb.2015.00815

**Published:** 2015-08-05

**Authors:** Viola C. Scoffone, Olga Ryabova, Vadim Makarov, Paolo Iadarola, Marco Fumagalli, Marco Fondi, Renato Fani, Edda De Rossi, Giovanna Riccardi, Silvia Buroni

**Affiliations:** ^1^Laboratory of Molecular Microbiology, Dipartimento di Biologia e Biotecnologie “Lazzaro Spallanzani,” Università degli Studi di PaviaPavia, Italy; ^2^Bakh Institute of Biochemistry, Russian Academy of ScienceMoscow, Russia; ^3^Department of Biology, University of FlorenceFlorence, Italy

**Keywords:** drug resistance, new antimicrobials, Gram-negatives, cystic fibrosis, eﬄux pumps, *Burkholderia*

## Abstract

*Burkholderia cenocepacia* is a major concern for people suffering from cystic fibrosis as it contributes to serious respiratory tract infections. The lack of drugs effective against this opportunistic pathogen, along with the high level of resistance to multiple antibiotics, render the treatment of these infections particularly difficult. Here a new compound, belonging to the 2,1,3-benzothiadiazol-5-yl family (10126109), with a bactericidal effect and a minimal inhibitory concentration (MIC) of 8 μg/ml against *B. cenocepacia*, is described. The compound is not cytotoxic and effective against *B. cenocepacia* clinical isolates and members of all the known *B. cepacia* complex species. Spontaneous mutants resistant to 10126109 were isolated and mutations in the MerR transcriptional regulator BCAM1948 were identified. In this way, a mechanism of resistance to this new molecule was described, which relies on the overexpression of the RND-9 eﬄux pump. Indeed, *rnd-9* overexpression was confirmed by quantitative reverse transcription PCR, and RND-9 was identified in the membrane fractions of the mutant strains. Moreover, the increase in the MIC values of different drugs in the mutant strains, together with complementation experiments, suggested the involvement of RND-9 in the eﬄux of 10126109, thus indicating again the central role of eﬄux transporters in *B. cenocepacia* drug resistance.

## Introduction

*Burkholderia cenocepacia*, together with *B. multivorans*, contributes to serious respiratory tract infections in people suffering from cystic fibrosis (CF; LiPuma, 2010). Worldwide about 80000 people are affected by CF, with a life expectancy of about 50 years; a proven effective therapy aimed at correcting the basic genetic defect is still lacking (Dodge et al., 2007). The disease causes the formation of thick and sticky mucus especially in the lungs, leading to serious infections that in 2009 in Europe accounted for the 45% of CF deaths^1^. In particular, *B. cenocepacia* is one of the worst CF-associated pathogens, as it carries the greatest risk of mortality, and it can lead to a fatal pneumonia called the “*cepacia* syndrome.” Moreover, the low percentage of post-transplantation survival among *B. cenocepacia* infected patients makes the infection due to this bacterium a contraindication for transplantation in most hospitals (Noone, 2008; De Soyza et al., 2010). Finally, due to its high level of resistance to most antibiotics (aminoglycosides, polymyxins, and β-lactams) it is particularly difficult to eradicate (Saiman et al., 2003). Accordingly, treatment of *B. cenocepacia* infections is often unsuccessful and triple antibiotic combination therapy is only aimed at decreasing bacterial load (Aaron et al., 2000).

Active transport is now recognized as a major cause of antimicrobial resistance in bacteria (Higgins, 2007). In *B. cenocepacia* the transporters belonging to the RND (Resistance-Nodulation-Cell Division) family are encoded by 16 operons (Perrin et al., 2013). We previously showed, by gene inactivation, the importance of RND-3 (BCAL1674-76) and RND-4 (BCAL2820-22) in the intrinsic resistance of *B. cenocepacia* J2315 as well as their involvement in the accumulation of quorum sensing molecules in the medium (Buroni et al., 2009). More recently, RND-4 has also been shown to be responsible for the resistance to a new antitubercular thiopyridine compound effective against *B. cenocepacia* (Scoffone et al., 2014). In another recent work, the effect of the deletion of the 16 RND operons was evaluated both in planktonic and sessile cells (Buroni et al., 2014). In particular, RND-3 and RND-4 have been shown to play a major role in the resistance to various antimicrobial drugs in planktonic *B. cenocepacia* J2315, while RND-3, RND-8, and RND-9 were needed to protect sessile cells against tobramycin or ciprofloxacin (Buroni et al., 2014).

In this study, a new molecule effective against *B. cenocepacia* J2315 is described. The compound belongs to the 2,1,3-benzothiadiazol-5-yl family and was named 10126109. A mechanism of resistance to this new molecule is described, which relies on the overexpression of the RND-9 eﬄux pump, thus indicating, once again, the central role of eﬄux transporters in *B. cenocepacia* drug resistance.

## Materials and Methods

### Bacterial Strains and Growth Conditions

*Burkholderia cenocepacia* strains and *B. cepacia* complex (Bcc) species were grown in Luria-Bertani (LB) medium (Difco), with shaking at 200 rpm, or on LB agar, at 37°C. *B. cenocepacia* J2315 was used as the wild-type (WT) strain.

### Synthesis of Methyl [(4-nitro-2,1,3-benzothiadiazol-5-yl)thio]acetate (10126109)

Solution of 1.43 g (10 mmol) of 2-amino-4-chloroaniline in the mixture of 5.4 ml thionyl chloride and 0.35 ml of concentrated sulfuric acid was refluxed for 1 h. The reaction mixture was cooled to 35°C, mixed with 4.3 ml of concentrated sulfuric acid and stored for 20 min. This solution was treated by mixture of 1.43 ml (32 mmol) fumigating nitric acid and 2.2 ml concentrated sulfuric acid at 20–25°C for 10 min. The reaction mixture was stored at 25–30°C for 30 min and poured to ice water. Gray precipitate was filtered off, washed by water and methanol and recrystallised from methanol. The yield of 5-chloro-4-nitro-2,1,3-benzothiadiazole is 1.6 g (74%). mp: 145–147°C.

Suspension of 0.5 g (2.32 mmol) of 5-chloro-4-nitro-2,1,3-benzothiadiazole, 0.32 ml (3.5 mmol) of methyl mercaptoacetate and 0.45 g (3.5 mmol) of potassium carbonate in 25 ml of acetonitrile was stored for 30 min at room temperature. The reaction mixture was poured in 100 ml of cold water and yellow precipitate was filtered off. The yield of methyl [(4-nitro-2,1,3-benzothiadiazol-5-yl)thio]acetate is 0.17 g (24%). mp: 121–123°C (methanol); MS (m/z): 285 (M^+^); ^1^H NMR (DMSO-d_6_): δδ 8.22 (1H, d, *J* = 8.1 Hz, CH), 7.67 (1H, d, *J* = 8.8 Hz, CH), 3.89 (2H, s, CH_2_), 3.71 (3H, s, CH_3_) ppm. Anal. C_9_H_7_N_3_O_4_S_2_, C,H,N.

### Determination of Cytotoxicity

HeLa Ohio cells were seeded at 2.4 × 10^4^ cells/well in 96-well flat-bottomed microtiter plates. To determine the 50% cytotoxic concentration (CC_50_), 2-days-old confluent HeLa cell monolayer were incubated with serial dilutions (factor 2, each concentration in duplicate) of the 10126109 compound for 72 h (37°C, 5% CO_2_). Then, the cells were fixed and stained with a crystal violet formalin solution. Cytotoxicity was quantified spectrophotometrically with a plate reader as described previously (Schmidtke et al., 2001).

The potential mutagenicity was assessed using the SOS-chromotest at 50 μM as previously described (Quillardet et al., 1982).

### Minimal Inhibitory Concentrations (MIC) Determination

The effectiveness of 10126109 compound against *B. cenocepacia* J2315, *B. cenocepacia* clinical isolates and Bcc species, was assessed determining MICs. The experiment was performed with the twofold microdilution method in U-bottom 96-well microtiter plates, and inoculating about 10^5^ CFU in LB medium, using concentrations ranging from 1 to 256 μg/ml. The microtiter plates were incubated at 37°C for 2 days and growth was determined by the resazurin method (Martin et al., 2006). A solution of resazurin sodium salt (Sigma–Aldrich) was prepared at 0.01% in distilled water and filter-sterilized. 30 μL of resazurin solution were added to each well after 2 days of incubation at 37°C, and the microtiters were reincubated at the same temperature for about 4 h. The MIC was defined as the lowest concentration of the drug that prevented a change in color from blue to pink, which indicates the growth of bacteria.

The same results were confirmed also by streaking 1 × 10^4^ cells onto LB agar containing twofold dilutions of the drug.

To obtain the resistance profile of *B. cenocepacia* mutants, the following compounds, at concentrations ranging from 2 to 256 μg/ml, were tested: chloramphenicol, ciprofloxacin, levofloxacin, nalidixic acid, norfloxacin, and sparfloxacin. All antibiotics were purchased from Sigma–Aldrich. In all experiments the results represent the average of three independent replicates.

### Time-Killing Curve

Time-killing curve of *B. cenocepacia* J2315 toward 10126109 was performed by the broth macrodilution method, as described by Silva et al. (2011). Briefly, 0.5, 1, 2, and 4 multiples of the MIC (8 μg/ml) were used. 20 ml of LB broth with the appropriate 10126109 concentrations were inoculated with exponentially grown *B. cenocepacia* cells, to yield a final concentration of approximately 1 × 10^7^ CFU per ml. The cultures were incubated at 37°C, and aliquots were removed at 0, 2, 4, 6, 8, 24, 28, and 32 h for the determination of viable counts. Serial dilutions were spread on LB solid medium and the plates were incubated at 37°C for 2 days. Then the number of colonies was determined.

Killing curves were constructed by plotting the log10 CFU ml^-1^ vs. time. Bactericidal activity was defined as a reduction of 99.9% (≥3 log10) of the total number of CFU ml^-1^ in the original inoculum (NCCLS, 1999). Bacteriostatic activity was defined as maintenance of the original inoculum concentration or a reduction of less than 99.9% (<3 log10) of the total number of CFU ml^-1^ in the original inoculum.

### Isolation of *B. cenocepacia* Mutants Resistant to 10126109

*Burkholderia cenocepacia* resistant mutants were isolated by plating about 10^9^ CFU of WT culture on LB agar, containing different concentrations of 10126109 compound, ranging from 4 to 20-fold the MIC. Plates were incubated at 37°C for at least 3 weeks. The resistance profile of each strain was confirmed by growing the colonies in liquid LB medium containing the same concentration of molecule at which the strains were isolated, and re-streaking the cells onto solid medium containing 10126109.

### Genomic DNA Extraction and Sequencing

Genomic DNA was extracted from *B. cenocepacia* J2315 and spontaneous mutants using Bacteria DNA Preparation Kit (Jena Bioscience). 10^9^ cells were harvested by centrifugation and treated as described in the manufacturer’s protocol. The DNA was resuspended in Tris-EDTA (TE) and used for sequencing purpose. Quality and concentration of each sample were assessed using spectrophotometer and agarose gel electrophoresis. All genomes were sequenced through Illumina Solexa technology (IGA Technology Service Srl). Reads quality was assessed using FastQC toolkit^2^. Reads trimming was performed using the dynamic trimming approach implemented in SolexQA (Cox et al., 2010), using a Phred score of 30 as the base call quality threshold. Trimmed reads were then mapped on the reference genome using Mosaik aligner with default parameters (Lee et al., 2014). Only those SNPs that (i) were not present in the WT strain, (ii) were supported by, at least, eight reads, and (iii) had a support greater than 70% were considered for further analysis. SNPs calling was performed using VarScan (Koboldt et al., 2009).

Mutations were confirmed by sequencing PCR amplicons using the following primer pairs with HotStar HiFidelity Polymerase kit (Qiagen): BCAM1948FOR (5′-ATGGGTATCCAGGAAACC-3′) and BCAM1948REV (5′-GAGTCGCTGCAGTTCCTCGA-3′) for SB5 and SB36 mutants; BCAM1948SB34F (5′-AAATCGCCGAGCAGCTCGTCAGC-3′) and BCAM1948SB34R (5′-AGCCGGACGACATCGCGGACGC-3′) for SB34 mutant; BCAM1948SB6F (5′-TAAACTGCCGCGTAGCAT-3′) and BCAM1948REV for SB6 mutant.

### RNA Purification and Reverse Transcription (RT)

For the Quantitative reverse transcription PCR (qRT-PCR) experiment, WT and mutant *B. cenocepacia* cells (1 × 10^9^ CFU) were collected by centrifugation. Total RNA was extracted using the RiboPure Bacteria Kit (Ambion), following the manufacturer’s instructions. A 30 min incubation of each sample with DNase I (Ambion) was performed, following the manufacturer’s protocol. RNA quality and concentration were assessed using both agarose gel electrophoresis and the spectrophotometric determination. One-microgram of total RNA was used for cDNA generation using the QuantiTect reverse Transcription kit (Qiagen) according to the manufacturer’s instructions, but diluting the cDNA 1:2 before using for qRT-PCR.

### Quantitative Reverse Transcription PCR (qRT-PCR)

For each strain, qRT-PCR experiments on *BCAM1946* gene were performed. The following primers were used: BCAM1946Rtfor (5′-TGCTCGTCGTGATCCTGTTT-3′) and BCAM1946Rtrev (5′-CGAACAGCGTGAGCGTATTG-3′). All reactions were performed on a Rotor-Gene-6000 cycler (Corbett), using Quanti-Tect SYBR Green PCR Kit (Qiagen), according to manufacturer’s instructions. Fifteen-microliter were used as a final volume for each reaction. Cycling conditions were: 95°C for 15 min (1 cycle), 94°C for 15 s followed by 54°C for 30 s and 72°C for 30 s (40 cycles). A melting curve analysis was included at the end of each run. Each sample was spotted in triplicate and a reference gene, as well as control samples without cDNA, were included in each experiment. *BCAM0918* (*rpoD*) gene was used as reference gene with the following primers: 0918F (5′-GCCAACCTGCGTCTCGT-3′) and 0918R (5′-AACTTGTCCACCGCCTT-3′), using an annealing temperature of 50°C. The fold difference in gene expression between the mutants and the WT was assessed using the comparative Ct-method (Livak and Schmittgen, 2001). The results are the average of three independent replicates. To determine if differences in expression were significant (*P* < 0.05) the Mann–Whitney test was used.

### Complementation

For complementation experiments the entire *BCAM1948* gene was PCR amplified from *B. cenocepacia* J2315 genomic DNA using primers 1948compF (5′-TTCATATGTAAACTGCCGCGTAGCAT-3′) and 1948compR (5′-TTGGATCCCGTCATTCGCAATCGGTC-3′). The resulting PCR product was digested with *Nde*I and *BamH*I (restriction sites are underlined in the primer sequences) and cloned into pSCrhaB2 (Cardona and Valvano, 2005; kindly provided by Prof. M. Valvano) that had been treated with the same restriction endonucleases.

The recombinant vector was then introduced into *B. cenocepacia* resistant mutants by conjugation via a triparental mating, as previously described (Craig et al., 1989). The expression of *BCAM1948* was induced by adding rhamnose (0.2%) to the LB medium. The MIC of 10126109, chloramphenicol, ciprofloxacin, levofloxacin, nalidixic acid, norfloxacin, and sparfloxacin were determined for both the mutant strains transformed with the empty vector and for the same strains carrying the BCAM1948/pSCrhaB2 vector, all grown in LB medium in the presence of trimethoprim (800 μg/ml) and 0.2% (w/V) rhamnose.

## Results

### Identification of a New Compound with Antimicrobial Activity Against *B. cenocepacia*

**FIGURE 1:**
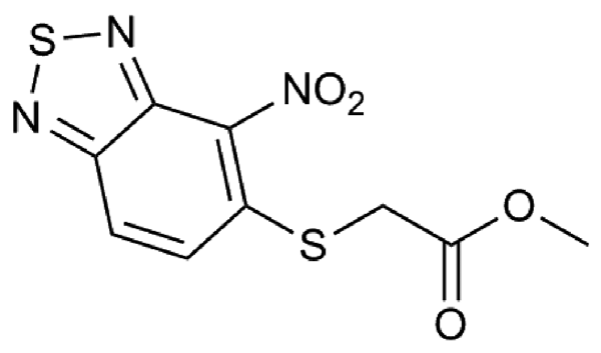
**Chemical structure of 10126109 compound**.

More than 100 compounds from an in-house collection and shown to be effective against other bacteria were tested for their efficacy against *B. cenocepacia* J2315. For each compound, the MIC was evaluated through the twofold microdilution method (Martin et al., 2006); only a compound belonging to the 2,1,3-benzothiadiazol-5-yl family (named 10126109, **Figure 1**), was found to have a promising antibacterial activity, showing a MIC of 8 μg/ml against *B. cenocepacia* J2315 (**Table 1**).

**Table 1:** Antimicrobial susceptibilities (μg/ml) of *Burkholderia cenocepacia* J2315, of 10126109 resistant mutants, and of the complemented mutants.

*B. cenocepacia* strains	Compound MIC (μg/ml)						
	10126109	CHL	CIP	LVX	NAL	NOR	SPX
J2315	8	32	4	4	16	16	8
SB5	128	64	32	32	256	128	64
SB6	128	64	32	32	256	128	64
SB34	256	64	32	32	256	128	64
SB36	256	64	32	64	256	128	128
SB5/pSCrhaB2	64	64	32	16	256	128	64
SB5/pSCrhaB2-1948	16	32	16	16	32	32	16
SB34/pSCrhaB2	64	64	32	16	256	128	64
SB34/pSCrhaB2-1948	8	32	8	8	32	32	16
SB36/pSCrhaB2	128	64	64	32	256	128	64
SB36/pSCrhaB2-1948	8	32	8	8	32	32	16

*CHL, chloramphenicol; CIP, ciprofloxacin; LVX, levofloxacin; NAL, nalidixic acid; NOR, norfloxacin; SPX, sparfloxacin.*

Cytotoxicity of the lead compound 10126109 was determined on HeLa cells and shown a 50% cytotoxic concentration (CC_50_) > 100 μM. The compound was also tested for its potential mutagenicity using the SOS-chromotest at 50 μM and the test was negative. 10126109 has good pharmacokinetic properties, low toxicity and a scaffold already used in commercial compounds (e.g., Tizanidine hydrochloride).

**FIGURE 2:**
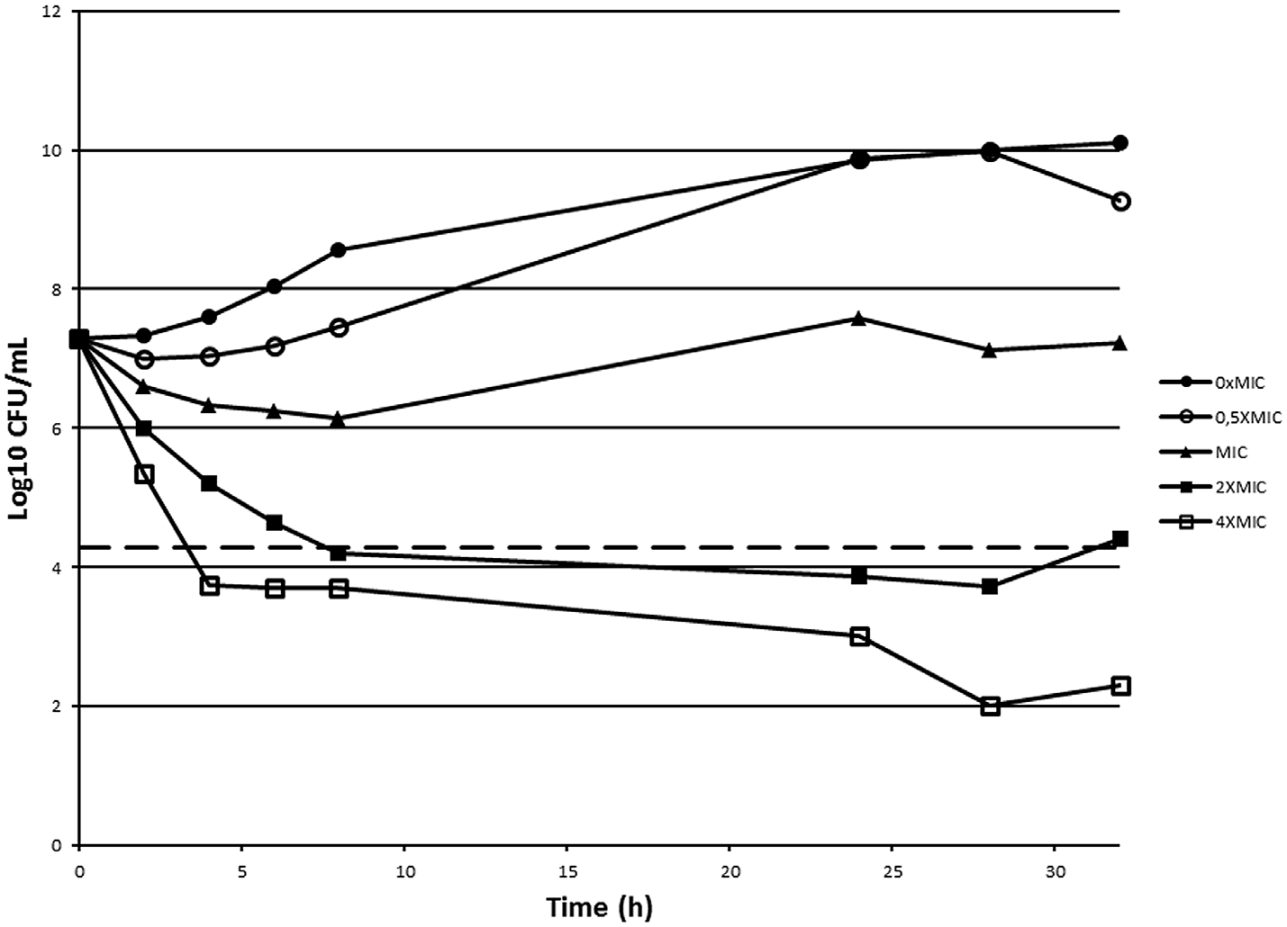
**Time-kill curve of *Burkholderia cenocepacia* J2315 exposed to 10126109.** A *B. cenocepacia* J2315 culture in exponential phase of growth (∙) was split and concentrations of 10126109 corresponding to 0.5 (○), 1 (▴), 2 (■), and 4-fold (□) the MIC value were added. The viable counts were determined at 37°C during 32 h. The dotted line indicates a reduction of 3 log10.

In order to assess if 10126109 exerts a bacteriostatic or a bactericidal effect on *Burkholderia* cells, a time-kill curve was constructed using concentrations ranging from 0.5 to 4 times the MIC value (**Figure 2**). A bactericidal effect was observed using two and fourfold the MIC of 10126109 (i.e., a reduction of 99.9% (≥3 log10) of the total number of CFU ml^-1^ in the original inoculum was observed), while the number of *B. cenocepacia* cells remained constant when an amount of compound equal to the MIC was added to the culture. These data suggest that bacterial killing is concentration dependent, as already reported for ciprofloxacin (Silva et al., 2011).

To assess if 10126109 is active against clinical circulating strains, the compound was tested against 30 *B. cenocepacia* clinical isolates (belonging to genomovars III-A, III-B, and III-D). All the strains resulted to be susceptible to 10126109, with MIC values ranging from <2 to 32 μg/ml, except three strains belonging to genomovar III-B, which showed MICs of 64–256 μg/ml (Supplementary Table S1). These data provide a good starting point for pre-clinical trials.

Subsequently, the compound was tested also against members of all the other Bcc species (Supplementary Table S2), showing MIC values ranging from 8 μg/ml (*B. ambifaria, B. anthina, B. arboris*, and *B. lata*) to 32 μg/ml (*B. stabilis, B. ubonensis*, and *B. vietnamiensis*).

### Isolation and Characterization of Spontaneous Mutants Resistant to 10126109

To isolate spontaneous resistant mutants in an attempt to find the 10126109 target(s), *B. cenocepacia* J2315 cells were spread on plates containing 4–20-fold the MIC of the compound.

Two spontaneous mutants isolated through direct selection onto solid medium containing 64 μg/ml of the benzothiadiazol derivative (eightfold the MIC), and two additional mutants, isolated from plates containing LB plus 128 μg/ml of the compound (16-fold the MIC) were selected for further characterization. These mutant strains were named SB5, SB6, SB34, and SB36, respectively, and the resistance phenotype was confirmed for all the mutants (**Table 1**). The mutation frequency was about 4 × 10^-9^.

The genomic DNA of mutant strains was then extracted and sequenced by Illumina Solexa method (IGA Technology Services, Udine) with the aim to understand which mutations were responsible for the resistance profile. The only gene resulting mutated in all the strains was *BCAM1948*. It encodes a 159 amino acid protein, which belongs to the MerR family of transcriptional regulators. As it is located immediately downstream from the *BCAM1945–BCAM1947* operon, encoding the putative quinoxaline eﬄux system transporter, previously named RND-9 (Bazzini et al., 2011), we hypothesized that this protein controls the expression of the eﬄux system.

The mutation identified in SB5 strain was the substitution C58T causing arginine-20 to be replaced by a cysteine residue. In the SB36 resistant mutant, C-58 was changed into an A, leading to the replacement of arginine-20 with a histidine residue.

In SB6 mutant, the mutation was located in the intergenic region between *BCAM1947* and *BCAM1948*, where A-49 is deleted. Interestingly, this deletion is located in a palindromic sequence (5′-ttgaagttaacttcaa-3′).

In SB34 mutant, the mutation was the substitution T380C, which led to the amino acid change Leu-153→Pro, having the two amino acids completely different chemical properties.

PCR amplification and sequencing were performed as described in Section “Materials and Methods” and confirmed the presence of the respective mutations in the genome of the four mutant strains.

Furthermore, we analyzed the evolutionary conservation and the possible functional consequences of the mutations falling within the coding region of *BCAM1948*. First, we checked the conservation of Arg-20 and Leu-153 in closely related microbes by aligning the best 30 BLAST hits (probing the NCBI non-redundant database) of the *BCAM1948* encoded protein. A close inspection of this multialignment revealed that the Arg-20 was conserved throughout all the analyzed genomes (see Supplementary Figure S1), suggesting the presence of some functional constraints acting in this position. Next, we checked the location of the mutated amino acids on the three-dimensional structure of the encoded protein. A search in Swiss Model Expasy (Biasini et al., 2014), revealed that the closest structural template of the MerR–like transcriptional regulator encoded by *BCAM1948* is the (2Fe-2S) oxidative-stress sensor SoxR from *Escherichia coli* (STML id: SMTL id: 2zhh.1, Watanabe et al., 2008). The region (aa 1–80) embedding this residue (which resulted to be located at the accessible molecular surface) is predicted to be a DNA-binding domain. Interestingly, it has been shown that changes in the residues embedded in this region may lead to changes in the relative positions of all their protein subunits (5-helix, the DNA-binding domain and the Fe-S binding domain) and that the proper arrangement of the DNA-binding domain is essential for correct redox signal transduction (Watanabe et al., 2008). In particular, a change identical to the one identified in *B. cenocepacia* J2315 in this work (Arg-20→Cys) has been already observed in *E. coli* (Hidalgo et al., 1997), where it leads to an altered redox phenotype. Accordingly, we here speculate that the most likely consequence of the changes in the Arg-20 position in *B. cenocepacia* J2315 mutants is an impaired redox activity of the *BCAM1948* encoded protein.

Unfortunately, the other mutation in *BCAM1948* (Leu-153→Pro) falls in a region showing no homology with other known 3D structures of MerR-like proteins and thus it is not possible to infer any feature of the altered phenotype resulting from this mutation in *B. cenocepacia* J2315.

### Expression Analysis of RND-9

As the mutations responsible for the resistance to 10126109 were located in the gene encoding the transcriptional regulator BCAM1948, to determine whether this phenotype was associated with a differential expression of RND-9 system, quantitative RT-PCR experiments were performed. The expression levels of *BCAM1946*, which codes for the RND transporter portion of RND-9, were assessed. Data obtained revealed that *rnd-9* gene was greatly and significantly up-regulated in all the mutants (*P* < 0.0001) in respect to the WT strain, the overexpression ranging between 200-fold in SB6 and about 1000-fold in SB36 (**Table 2**).

**Table 2:** *BCAM1946* expression levels by quantitative reverse transcription PCR (qRT-PCR) in *B. cenocepacia* WT (J2315) and mutated strains.

*B. cenocepacia* strains	*BCAM1946* fold-change (±SD)
J2315	1.16 ± 0.85
SB5	855.97 ± 12.58
SB6	205.07 ± 16.30
SB34	469.22 ± 81.30
SB36	993.66 ± 72.99

In order to confirm that the *BCAM1946* overexpression established by qRT-PCR was related to the production of the corresponding protein, the membrane fractions of the *B. cenocepacia* WT and mutant strains were extracted and analyzed. The proteins present in this cellular fraction were analyzed by SDS-PAGE and Coomassie blue staining, as described in Supplementary data. A difference in the band pattern was observed between the WT and the four mutants, where a band of about 170 kDa was more abundant in the mutants in respect to the J2315 strain (Supplementary Figure S2). This difference was confirmed by repeating the experiment with independent cultures. The band was excised from the acrylamide gel and identified by mass spectrometry. The band was identified as BCAM1946, the inner membrane component of RND-9 (Supplementary Table S3), which was shown to be overexpressed by qRT-PCR.

### Antibiotic Susceptibility of Mutant Strains and of the RND-9 Deleted Strain

It is noteworthy that the overexpression of eﬄux pumps is responsible for a resistance phenotype in bacteria, being these transporters able to extrude different compounds. In this way, the MIC of different antibiotics was assessed for the four 10126109 resistant strains. The mutant strains showed a 2–16-fold increase in resistance toward all the compounds tested (**Table 1**). In particular, all of them were twofold more resistant to chloramphenicol, eightfold more resistant to ciprofloxacin, levofloxacin, norfloxacin, and sparfloxacin, and 16-fold more resistant to nalidixic acid compared to the WT strain (**Table 1**). SB36 mutant was 16-fold more resistant to levofloxacin and sparfloxacin (**Table 1**).

Although our previous finding suggested no correlation between RND-9 and ciprofloxacin resistance in *B. cenocepacia* (Buroni et al., 2014), being BCAM1948 a MerR transcriptional regulator, it could control the expression of other genes encoding both eﬄux pumps and/or involved in ciprofloxacin resistance.

To further assess whether RND-9 could be involved in 10126109 extrusion, the MIC of the compound was assessed also for the RND-9 deleted strain. In this case, the MIC was twofold lower respect to the WT.

Taken together these results confirmed that the RND-9 overexpression is responsible for resistance to 10126109, and suggested the involvement of this transporter in the 10126109 eﬄux.

### Complementation

To finally show that the mutations found in *BCAM1948* gene were responsible for the resistant phenotype of the four *B. cenocepacia* mutants, SB5, SB34, and SB36 strains were transformed by triparental conjugation with pSCrhaB2 vector carrying a WT copy of *BCAM1948*. The expression of *BCAM1948* was induced by adding 0.2% rhamnose to the growth medium (Cardona and Valvano, 2005).

Subsequently, the MIC of the compounds to which the four strains showed resistance, was assessed for the complemented strains (**Table 1**). The values were compared to the mutants transformed with the empty vector. Indeed, all the complemented strains showed MIC values (including those of 10126109) comparable to the *B. cenocepacia* J2315 strain (**Table 1**), indicating that complementation occurred.

Also these data confirmed the involvement of RND-9 in eﬄux-mediated resistance to 10126109.

These results, together with the overexpression of BCAM1946 shown by quantitative RT-PCR and membrane fraction extraction, indicate that BCAM1948 acts as a repressor for RND-9. In fact, in the presence of a mutated regulator (strains SB5, SB34, and SB36) or of an altered putative operator region (strain SB6) the expression of the eﬄux pump was greatly increased. It is noteworthy that the mutations located in the repressor coding region lead to a greater overexpression of RND-9 compared to the mutation located in the operator (SB6).

The MerR regulators have been originally described as activators (Brown et al., 2003), even if a different role has been hypothesized and a double function previously reported (Lee et al., 1993). Our results strongly suggest that MerR-like regulators can act as repressors in *B. cenocepacia*, in agreement with a recent work revealing their role as repressors in *Pseudomonas aeruginosa* (Chambers and Sauer, 2013).

## Discussion

It is noteworthy that unfortunately pharmaceutical industry is not investing in drug discovery against neglected infections such as those caused by *B. cenocepacia*, a dangerous CF pathogen. CF is a rare disease, and *B. cenocepacia* infections only occur in the 1–3% of patients. In this context, finding new molecules active against *B. cenocepacia* is of great importance as it is able to cause fatal infections which also lead the patient not to be admitted in transplant lists (Noone, 2008; De Soyza et al., 2010).

In this work, a compound belonging to the 2,1,3-benzothiadiazol-5-yl family has been described as a promising bactericidal anti-*Burkholderia* agent, being the MIC against *B. cenocepacia* J2315 8 μg/ml. The compound has been shown to be effective also against *B. cenocepacia* clinical isolates and Bcc belonging species, thus providing a good starting point for pre-clinical trials. It is noteworthy that the compound is not cytotoxic, nor mutagenic, suggesting its potential safety for future clinical trials on humans. The chemical structure of this compound will be useful also for future research and structure-activity relationship studies.

Even if there is not a formal eradication protocol for Bcc infections, often combinations of two or three drugs are administered, including nebulised tobramycin (MIC = 256 μg/ml) with or without additional oral antibiotics, such as minocycline (MIC = 16 μg/ml) or meropenem (MIC = 64 μg/ml; Horsley et al., 2011). EUCAST guidelines established that clinicians must continue to assess each patient individually and that it is not currently possible to establish MIC breakpoints for Bcc organisms^3^. In this context, the availability of a new chemical structure, showing an MIC lower respect to the currently used drugs, is a good and fundamental starting point to work on, in order to find new therapeutic solutions.

A mechanism of resistance to this new compound, which relies on the overexpression of RND-9 eﬄux pump, has been disclosed. Evidences came from the whole genome sequences of four resistant strains, showing mutations in the MerR transcriptional regulator BCAM1948 and responsible for the overexpression of the eﬄux pump both at the transcriptional and translational levels. Moreover, MIC determination of different compounds plus the complementation of the mutated strains with a WT copy of the gene confirmed the prominent role of the eﬄux pump in the resistance.

Our data support our previous studies on the role of *B. cenocepacia* eﬄux transporters in drug resistance (Buroni et al., 2009, 2014; Bazzini et al., 2011; Coenye et al., 2011; Scoffone et al., 2014). Among 16 eﬄux systems encoded in the genome of *B. cenocepacia*, only a few appear responsible for a resistant phenotype (RND-3, RND-4, RND-9, and RND-10). In this way, the use of new eﬄux inhibitors able to block these specific pumps coupled to new drugs able to interfere with *B. cenocepacia* growth, in order to avoid their extrusion, seems of primary importance in order to fight a main concern in Gram-negatives.

## Conflict of Interest Statement

The authors declare that the research was conducted in the absence of any commercial or financial relationships that could be construed as a potential conflict of interest.

## Footnotes

1 https://www.ecfs.eu/projects/ecfs-patient-registry/annual-reports

2 http://www.bioinformatics.babraham.ac.uk/projects/fastqc/

3 http://www.eucast.org/fileadmin/src/media/PDFs/EUCAST_files/General_documents/BCC_susceptibility_testing_130719.pdf
