# Supplementary material for: Eﬄux-mediated resistance to a benzothiadiazol derivative effective against Burkholderia cenocepacia
Source: Front Microbiol. 2015 Aug 5;6:815. doi: 10.3389/fmicb.2015.00815 (PMC4525489; doi:10.3389/fmicb.2015.00815)
Supplement: Supplementary file 1 [file Data_Sheet_1.DOCX]

***Supplementary Material***

**Efflux-mediated resistance to a benzothiadiazol derivative effective against *Burkholderia cenocepacia***

**Viola C. Scoffone^1^, Olga Ryabova^2^, Vadim Makarov^2^, Paolo Iadarola^1^, Marco Fumagalli^1^, Marco Fondi^3^, Renato Fani^3^, Edda De Rossi^1^, Giovanna Riccardi^1^, Silvia Buroni^1*^**

^1^Dipartimento di Biologia e Biotecnologie L. Spallanzani, Università degli Studi di Pavia, Pavia, Italy

^2^Bakh Institute of Biochemistry, Russian Academy of Science, Moscow, Russia

^3^Department of Biology, University of Florence, Sesto Fiorentino (Florence), Italy

*** Correspondence:** Silvia Buroni: silvia.buroni@unipv.it

1. **Supplementary Data**

**Preparation of membrane fractions and SDS-PAGE**

*B. cenocepacia* membrane fractions were prepared as described by Biot *et al*. (Biot *et al*., 2013). Briefly, *B. cenocepacia* WT and mutant cells were grown until exponential phase (OD_600nm_ = 1) in LB medium and harvested by centrifugation. Cell pellets were lysed with 2 ml of lysis buffer (100 mM NaH2PO4/Na2HPO4 pH 7.4, 1 mg/ml lysozyme) and sonicated 5 times for 10 sec at 40% amplitude. Samples were then centrifuged for 10 min at 16000 × *g* at 4°C. The supernatants were centrifuged for 1 h at ~100000 × *g* at 4°C (Beckman Optima MAX-XP). Pellets were resuspended in 2 ml of 100 mM phosphate buffer pH 7.4 containing 0.15% N-laurylsarcosinate and incubated for 30 min at room temperature to extract the detergent-soluble material. Samples were centrifuged for 1 h at 100000 × *g* at 20°C. Pellets were then resuspended in 25 µl of sample buffer 4X (180 mM Tris-HCl pH = 8.0, 40% glycerol, 4% SDS, 0.04% bromophenol blue, 200 mM DTT) and heated for 5 min at 95°C. Samples were then run on 10% SDS-polyacrylamide gel. After migration, gels were stained with Coomassie Brilliant Blue G.

***In situ* enzymatic digestion**

The selected band was carefully excised from the gel slab, placed into eppendorf tubes and broken into small pieces. This material was washed twice with aliquots (200 µL) of 100 mM ammonium bicarbonate buffer pH 7.8, containing 50% acetonitrile (ACN) and kept under stirring overnight, until complete destaining. Gels were then dehydrated by addition of ACN (100 µL). After removing the organic solvent, reduction was performed by incubating samples for 40 min at 37°C with 10 mM Dithiothreitol (DTT, 50 µL). DTT was then replaced with 50 µL of 55 mM iodoacetamide and incubation prolonged for additional 45 min at 56°C. This solution was finally removed and the gel pieces were washed (twice) with 200 µL of 100 mM ammonium bicarbonate for 10 min, while vortexing. Further steps were: i) removal of the washing solution; ii) gel dehydration (by addition of 200 µL of ACN) until the gel pieces became an opaque-white color; iii) final removal of ACN and drying under vacuum of gel pieces. After rehydrating gels by addition of 75 µL of 100 mM ammonium bicarbonate buffer pH 7.8, 20 ng/µL of sequencing grade trypsin (Promega, Madison, WI, USA) were added and digestion was performed incubating overnight at 37°C. The resultant peptides were then extracted sequentially from gel matrix by treatment (at 37°C for 15 min) with: i) 50 µL of 50% ACN in water, ii) 5% trifluoroacetic acid and iii) 50 µL of 100% ACN. Each extraction involved 10 min of stirring followed by centrifugation and removal of the supernatant. The original supernatant and those obtained from sequential extractions were pooled, dried and stored at –80°C until mass spectrometric analysis. At the moment of use, the peptide mixture was solubilized in 100 µL of 0.1% formic acid (FA) for MS analyses.

**LC-MS/MS**

All analyses were carried out on an LC-MS (Thermo Finnigan, San Jose, CA, USA) system consisting of a thermostated column oven Surveyor autosampler controlled at 25°C; a quaternary gradient Surveyor MS pump equipped with a diode array detector and a Linear Trap Quadrupole mass spectrometer with electrospray ionization ion source controlled by Xcalibur software 1.4. Analytes were separated by reverse-phase high-performance liquid chromatography on a Jupiter (Phenomenex, Torrance, CA, USA) C18 column (150 x 2 mm, 4 µm, 90 Å particle size) using a linear gradient (2–60% solvent B in 60 min) in which solvent A consisted of 0.1% aqueous FA and solvent B of ACN containing 0.1% FA. Flow-rate was 0.2 mL/min. Mass spectra were generated in positive ion mode under constant instrumental conditions: source voltage 5.0 kV, capillary voltage 46 V, sheath gas flow 40 (arbitrary units), auxiliary gas flow 10 (arbitrary units), sweep gas flow 1 (arbitrary units), capillary temperature 200°C, tube lens voltage –105 V. MS/MS spectra, obtained by collision-induced dissociation studies in the linear ion trap, were performed with an isolation width of 3 Th m/z, the activation amplitude was 35% of ejection RF amplitude that corresponds to 1.58 V. Data processing was performed using Peaks studio 4.5 software. The mass lists were searched against the SwissProt protein database under continued mode (MS plus MS/MS) with the following parameters: trypsin specificity, five missed cleavages, peptide tolerance at 0.2 Da and MS/MS tolerance at 0.25 Da. Peptide charge 1, 2, 3+ and experimental mass values: monoisotopic.

1. **Supplementary Figures and Table**


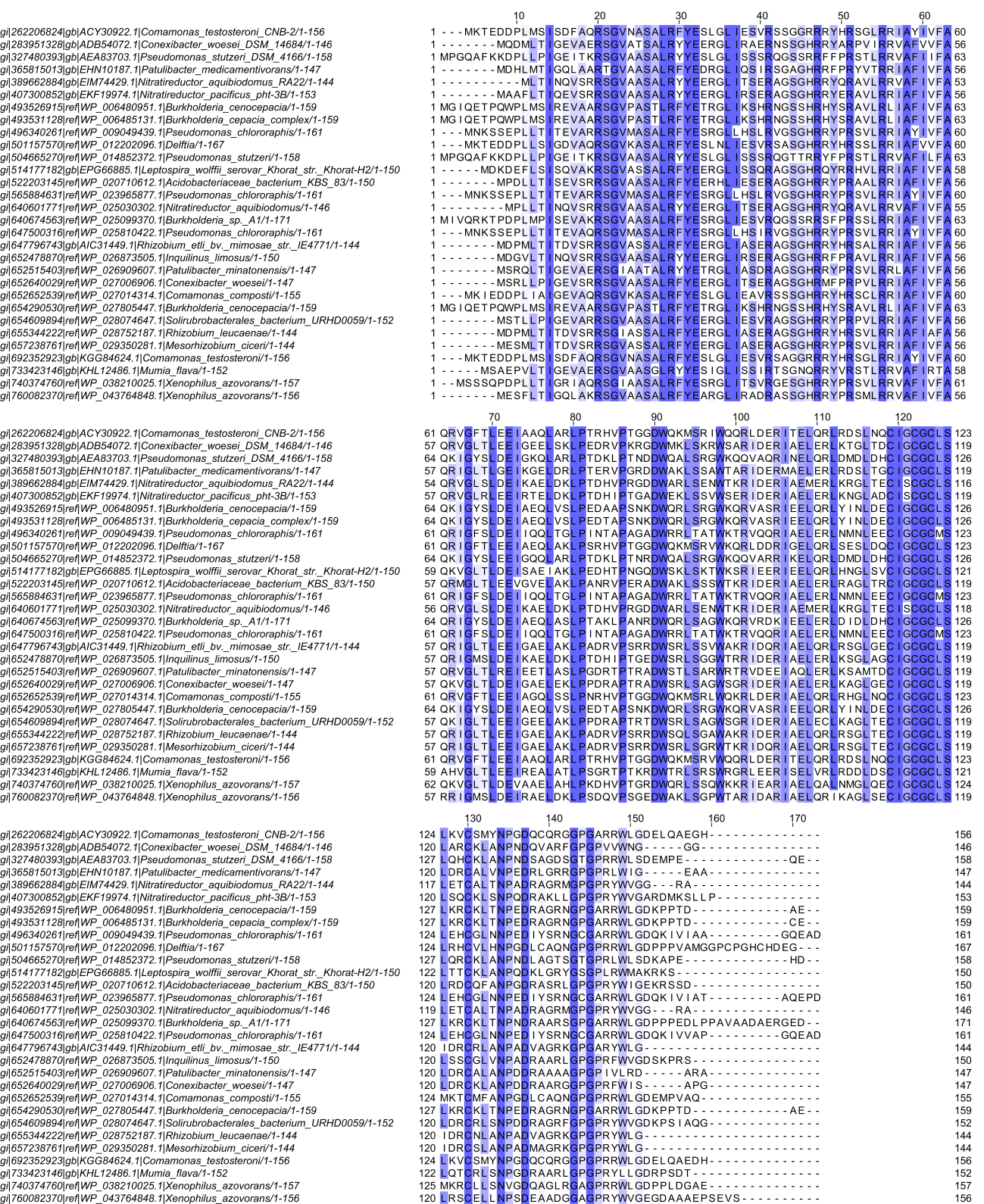


Supplementary Figure 1. Multialignment of the best 30 BLAST hits of the *BCAM1948* encoded protein. Conserved residues are highlighted in blue.


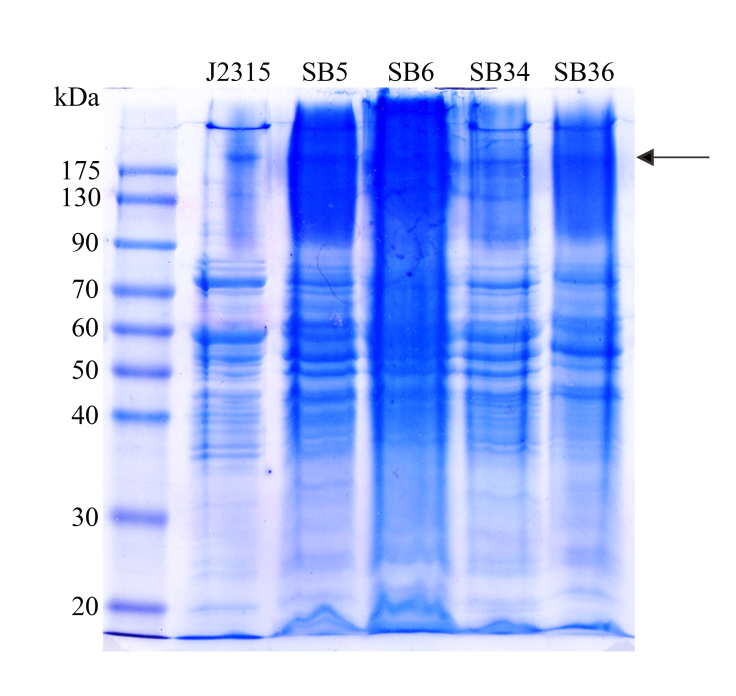


Supplemenatry Figure 2. Analyses of the detergent-insoluble membrane proteins of *B. cenocepacia* J2315 and of the resistant strains. SDS-PAGE analysis of the membrane fractions of the J2315 WT strain of *B. cenocepacia*, and of the four 10126109 resistant strains, SB5, SB6, SB34 and SB36 is shown. Proteins were stained with Coomassie blue. The overexpressed band at around 170 kDa is indicated by the arrow. Molecular weight standards (Nippon Genetics) are indicated in kilodaltons.

## Supplementary Tables

**Supplementary Table 1. Antimicrobial susceptibilities (µg/ml) of *B. cenocepacia* clinical isolates to 10126109.**

| ***B. cenocepacia* clinical isolates (genomovar)** | **Provenience** | **MIC (µg/ml)** |
| --- | --- | --- |
| 451 (III-A) | University Hospital Motol (Prague, Czech Republic) | **4** |
| 1140 (III-A) | University Hospital Motol (Prague, Czech Republic) | **4** |
| 1232 (III-A) | University Hospital Motol (Prague, Czech Republic) | **8** |
| 2560 (III-A) | University Hospital Motol (Prague, Czech Republic) | **8** |
| 3167 (III-A) | University Hospital Motol (Prague, Czech Republic) | **32** |
| 4742 (III-A) | University Hospital Motol (Prague, Czech Republic) | **32** |
| FCF 12 (III-A) | Papaleo *et al*. (2010) | **<2** |
| FCF 13 (III-A) | Papaleo *et al*. (2010) | **32** |
| FCF 14 (III-A) | Papaleo *et al*. (2010) | **8** |
| FCF 15 (III-A) | Papaleo *et al*. (2010) | **8** |
| FCF 16 (III-A) | Papaleo *et al*. (2010) | **4** |
| FCF 17 (III-A) | Papaleo *et al*. (2010) | **4** |
| 7016 (III-B) | University Hospital Motol (Prague, Czech Republic) | **32** |
| FCF 18 (III-B) | Papaleo *et al*. (2010) | **64** |
| FCF 19 (III-B) | Papaleo *et al*. (2010) | **128** |
| FCF 20 (III-B) | Papaleo *et al*. (2010) | **32** |
| FCF 21 (III-B) | Papaleo *et al*. (2010) | **8** |
| FCF 22 (III-B) | Papaleo *et al*. (2010) | **256** |
| FCF 23 (III-B) | Papaleo *et al*. (2010) | **16** |
| FCF 24 (III-B) | Papaleo *et al*. (2010) | **4** |
| FCF 25 (III-B) | Papaleo *et al*. (2010) | **16** |
| FCF 26 (III-B) | Papaleo *et al*. (2010) | **4** |
| FCF 27 (III-B) | Papaleo *et al*. (2010) | **4** |
| FCF 32 (III-D) | Papaleo *et al*. (2010) | **32** |
| FCF 33 (III-D) | Papaleo *et al*. (2010) | **16** |
| FCF 34 (III-D) | Papaleo *et al*. (2010) | **32** |
| FCF 36 (III-D) | Papaleo *et al*. (2010) | **16** |
| FCF 37 (III-D) | Papaleo *et al*. (2010) | **32** |
| FCF 38 (III-D) | Papaleo *et al*. (2010) | **16** |
| FCF 39 (III-D) | Papaleo *et al*. (2010) | **16** |

**Supplementary Table 2. Antimicrobial susceptibilities (µg/ml) of *Burkholderia cepacia* complex species to 10126109.**

| **Strain** | **MIC (µg/ml)** |
| --- | --- |
| ***Burkholderia cepacia* complex** |  |
| *B. ambifaria* LMG 19182 | **8** |
| *B. anthina* LMG 20980 | **8** |
| *B. arboris* LMG 24066 | **8** |
| *B. cenocepacia* LMG 16656 | **8** |
| *B. cepacia* LMG 1222 | **16** |
| *B. contaminans* LMG 23361 | **16** |
| *B. diffusa* LMG 24065 | **16** |
| *B. dolosa* LMG 18943 | **16** |
| *B. lata* LMG22485 | **8** |
| *B. latens* LMG 24064 | **16** |
| *B. metallica* LMG 24068 | **16** |
| *B. multivorans* LMG 13010 | **16** |
| *B. pyrrocinia* LMG 14191 | **16** |
| *B. seminalis* LMG 24067 | **16** |
| *B. stabilis* LMG 14294 | **32** |
| *B. ubonensis* LMG 20358 | **32** |
| *B. vietnamiensis* LMG 10929 | **32** |

**Supplementary Table 3. Protein identification through peptide digestion and mass spectrometry.**

| Accession | Mass | Score (%) | Description | Charge | Peptides |
| --- | --- | --- | --- | --- | --- |
| tr\|B4EMS9\|B4EMS9_BURCJ | 113,416 | 99 | Putative quinoxaline efflux system transporter protein OS=*Burkholderia cenocepacia* | 2 | LPEGASLAR |
|  |  |  |  | 2 | LASRGLTASDVIAAVR |
|  |  |  |  | 2 | SAAQINADLNAR |
|  |  |  |  | 2 | YHGVVGRTLKRR |
|  |  |  |  | 2 | EQNVQVSAGQLGAEPSPK |

1. **Supplementary References**

Biot, F.V., Lopez, M.M., Poyot, T., Neulat-Ripoll, F., Lignon, S., Caclard, A., *et al*. (2013). Interplay between three RND efflux pumps in doxycycline-selected strains of *Burkholderia thailandensis*. *PLoS One* **8**:e84068. doi: 10.1371/journal.pone.0084068

Papaleo, M.C., Perrin, E., Maida, I., Fondi, M., Fani, R., Vandamme, P. (2010). Identification of species of the *Burkholderia cepacia* complex by sequence analysis of the *hisA* gene. *J. Med. Microbiol.* **59**:1163-1170. doi: 10.1099/jmm.0.019844-0
